# Editorial

**Published:** 1862-04

**Authors:** 


### Editorial.

Postponement of the Annual Meeting of the Illinois State Medical Society.—In accordance with the instructions of the State Medical Society at its last meeting, the Secretary issued the usual notice for the Annual Meeting to be held in Jacksonville, on the first Tuesday in May next. Soon after the issuing of that notice, the Committee of Arrangements of the American Medical Association notified a postponement of the meeting of that Association until June, 1863. On examination of the records, it was found that the President, one Vice-President, Chairmen of most of the Committees, and a large proportion of the active members, were engaged as Brigade and Regimental Surgeons, on active duty with different divisions of the army; while the large number wounded in the recent battles at Fort Donelson and Pittsburg Landing, had called many more from their homes, temporarily, as volunteer surgeons. Under these circumstances, the Chicago Medical Society, at a recent meeting advised a postponement of the Annual meeting of the State Society for another year. Not willing to act on this advice alone, the Secretary addressed letters to the Committee of Arrangements, at Jacksonville, and to eight or ten active members of the Society in different parts of the State. And though no answers have yet been received from several of those addressed, enough has been learned to make it quite certain that a meeting could not be held the present spring, with any prospect of success or profit. Two of the Committee of Arrangements at Jacksonville, are among the absentees with the army, in such positions that they cannot attend any meeting of the Society, or participate in any arrangement for the entertainment of its members. With these facts, and the recommendation of the remaining members of the Committee of Arrangements, we feel authorized to state that no meeting of this

*State Medical Society* will be held the present year. Official notice to this effect will be issued to all whose names are on the list of members, in a few days. While we regret the interruption of the regular meetings of our State Society, we certainly admire the *patriotism*, self-sacrifice, and love of country, manifested by so large a proportion of its members.

---

METROPOLITAN HEALTH BILL OF NEW YORK.—For several years the Medical Profession of New York City and State, have been endeavoring to procure the passage of such laws regulating the Sanitary affairs of the city and harbor of New York, as would greatly lessen not only the annual mortality of that city, but would also lessen the danger of having infectious and contagious diseases passed directly through it to all parts of the country. There is scarcely a town between New York city and the Rocky Mountains, that is not interested in the sanitary regulations of that city and harbor. It being the great receiving reservoir of immigration and commerce, unless efficient sanitary laws are enacted and faithfully enforced, every infectious or contagious disease, either engendered in its own dense population or imported with the tide of foreign immigration, is liable to be distributed to every interior point holding either social or commercial intercourse with that city. We have repeatedly had cases of ship-fever, brought from immigrant ships directly through New York city over the whole length of Railway to Chicago, and have treated cases both in private practice and in the Mercy Hospital here, in which the forming stage of the fever was well marked before they left the ship in the harbor of New York. Small-pox is another disease that is frequently brought from New York, both by immigrants and merchants, to the interior towns and cities. Many years ago when we lived in a flourishing town in the interior of New York State, its trade and commerce was almost completely interrupted the greater part of one summer by the importation of a case of small-pox into it from New York city. It is hardly possible to estimate the importance to the whole country, of well devised and efficiently executed sanitary laws for New York city. Since a very early part of the present session of the New York Legislature, a Health Bill for that city, which we are informed has been carefully devised by the most enlightened members of the medical profession of New York, has been urged upon the attention of that body. But as on all similar occasions in previous Legislatures, the infernal desire to make every act of legislation subservient to some political or partisan purpose, is likely either to defeat the measure or so amend it as to destroy its most valuable features. How long will the American people continue to permit their public servants, whether in New York or Illinois—whether in Washington or Albany, to sacrifice human life, human health, and every other interest worthy of preservation on the altar of partisan politics and personal ambition? A legislative body which should assemble in any part of our country, and devote itself for six weeks exclusively to the work of making honest and necessary laws for the improvement of Society and the enforcement of impartial justice, and adjourn without meddling with anything outside of their legitimate business, would be a greater curiosity than the indefatigable BARNUM ever dreamed of.

---

RESERVE CORPS OF ARMY SURGEONS.—We see that in New York and some other States, the authorities have organized a corps of Volunteer Surgeons to be in readiness to enter upon active duty, temporarily, whenever a severe battle, like that of Pittsburg Landing or Fort Donelson, shall render aid to the regular army surgeons necessary. This is an excellent arrangement, and should be effected in this State. There are doubtless fifteen or twenty experienced and skillful surgeons in the State who would cheerfully offer their services as a volunteer corps, and hold themselves in readiness to meet any emergency, that might occur during the progrsss of the war. Such an arrangement would, not only, secure the services of the most competent physicians and surgeons whenever they were needed, but it would enable the proper authorities to know, at all times, precisely where, and to whom they could apply for help.

Personal.—Our colleague, Dr. E. Andrews, professor of Surgery, in the Medical Department of Lind University, had accepted the post of Surgeon to Col. J. D. Webster's Regiment of Illinois Artillery, and left for the division of the army under Gen. Grant, just in time to make his untiring energy and skill, available in aid of the many wounded in the great battle at Pittsburg Landing. None more faithful, skillful, and humane, could be found in this or any other State.

---

The London Lancet.—The March number of this long established, and valuable embodiment of English medical opinion and practice, has been promptly received from the publishers. It is, as usual, filled with useful lectures and valuable practical matter, gathered from the numerous Schools, Societies, and Hospitals of London.

---

Obituary.—Died, on the morning of the 5th inst., E. B. Rockwell, M. D., of this city, aged 34 years. Dr. Rockwell has been a resident of the city and a teacher in Bell, Bryant & Stratton's Commercial College for several years. Three or four years since he commenced the study of medicine, and pursued it with diligence, as a member of the class in the Medical Department of Lind University. At the close of the last lecture term, only one month before his death, he graduated with credit to himself and the University. He was immediately employed as an assistant physician at Camp Douglas, and placed in charge of two hospital wards filled with the rebel prisoners, then, just received from Fort Donelson. After faithfully and skillfully discharging his duty to the sick prisoners for several weeks, during which he was much exposed to cold and wet, he was attacked severely, with pneumonia, chiefly limited to the middle and lower lobes of the left lung. From this he recovered in a few days, but imprudently exposed himself, both to cold and too much exercise, by re-visiting the camp and transacting some business about the streets of the city. He was again violently attacked with pleuro-pneumonia, which soon became complicated with an exhausting typhoid diarrhœa, and terminated fatally on the ninth day. Dr. Rockwell, was not only an excellent student, and skillful physician, but a highly esteemed citizen, an affectionate husband, and a most exemplary Christian. The place he held in the affections of the faculty and class of the medical department of the University, is truthfully indicated by the following resolutions, viz:

"RESOLUTIONS OF RESPECT.

At a meeting of the students of the medical department of the Lind University, held April 5th, the following resolutions were framed and adopted:

Whereas, An all wise Providence has in His wisdom removed from earthly labors, to heavenly joys, our friend and late fellow student in College, Dr. E. B. Rockwell, in the vigor of his manhood, and at the opening of a professional life full of promises of usefulness and honor, fills our hearts with the most profund sorrow.

*Resolved*, That we, his fellow students and companions, bow in meek submission to the mysterious doings of the Lord, in this bereavement; and, as a slight testimonial of our appreciation of his many virtues, will endeavor to imitate his worthy example.

*Resolved*, That in his decease, society has lost a worthy member; the church one of its brightest lights, as a christian; our profession, a noble, working brother; his family a beloved and loving husband and father; the world a true man.

*Resolved*, That we will cherish sacredly his memory, never forgetting his motto by which he was ever guided in action, "Nearer, my God, to Thee—nearer to Thee!"

*Resolved*, That his bereaved widow and friends have our heartfelt sympathy; and did we dare intrude on the sanctity of their sorrow, we should gladly approach them in this their time of sore affliction, and offer consolation, mingling our sympathizing tears of deep, inconsolable grief.

*Resolved*, That the family be furnished with a copy of these resolutions, and our city papers be requested to publish them.

John Quirk, President.

Alique C. Matchett, Secretary."

---

Paralysis.—The following, on the subject of Paralysis, as a sequel to diptheria, is taken from the *Boston Medical* and *Snrgical Journal.* The subject is one of interest, and though we have not observed it in so large a proportion of cases as stated by Dr. Roger, yet it is sufficiently frequent to demand attention. In the treatment of this form of paralysis, we have found nothing more promptly beneficial than a combination of strychnine and citrate of iron, in doses suited to the age of the patient.—*Ed. of Examiner.*

"Paralysis following Diptheria.—The paralysis of diptheria seems as yet to have attracted but little notice in this country, at least little has been published concerning it. Even in Europe but slight mention has been made of it in the various treatises on this disease which have been given to the world, although cases have appeared from time to time in the medical journals, so that the medical mind has become tolerably familiar with this sequela of a disease which, with the exception, perhaps, of Asiatic cholera, has engaged the thoughts of the profession and excited the apprehensions of the community of late years more than any other. M. Maingault has recently published in Paris what is said to be the first complete account of diphtheritic paralysis, in a pamphlet of 161 pages. We have not seen the work, but from the notice of it in the *British and Foreign Medico-Chirurgical Review* we take the following extract:—

'The outline which M. Maingault has sketched of the disease coincides entirely with its characters as they have presented themselves in this country. Thus, he has observed that the accession of the first symptoms usually takes place two or three weeks after the cessation of the primary local disease and the complete establishment of convalescence. In those cases in which the affection is about to attain its full development, the patient, instead of recovering his strength, begins gradually to become weaker; he experiences fornications in his extremities, usually first in the feet and legs, attended with or followed by varying degrees of numbness and insensibility, and gradually becomes unable to walk. As the paralysis attacks the upper limbs, and the disease progresses, vision is impaired or lost, the articulation becomes indistinct, and the voice nasal and weak; the constitutional state being marked by the absence of pyrexia, the feebleness of the pulse, the palor of the countenance, and the general characters of anæmia. There is often complete anorexia, but occasionally the appetite is preserved. The duration of this condition is various, it usually diminishes gradually, the occasionally fatal result being dependent either on gradual prostration or sudden asphyxia.'

M. H. Roger, in a paper read to the Hospital Medical Society in Paris, has discussed the same subject, to determine the frequency of the occurrence of these symptoms, and whether they are peculiar to pure, idiopathic diptheria. His observations were made on all the cases of this disease which were under treatment in the Children's Hospital during the year 1861. These were in number 210; and after deducting some doubtful cases, it was found that these symptoms occurred in 31 cases, or about one sixth of the whole number. Considering the fact that a certain proportion of the cases left the hospital before the disease had fully run its course, and others were prematurely cut off before the full development of all the symptoms likely to occur in grave cases' M. Roger thinks he is warranted in concluding that the ratio should be estimated as high as one fourth, or even one third, instead of one sixth. The paralytic symptoms showed themselves most frequently in children from 4 to 6 years of age, and in 21 females to 17 males.

The symptoms appeared, for the most part, first in the pharynx and velum palati, as was shown by the nasal character of the voice or the dysphagia. Often—four times in ten—it reached the lower extremities or became general, appearing first in the lower limbs and then in the upper in four cases out of ten. The same symptoms may appear in the course of cutaneous diphtherite, without any throat affection.

Paralysis of the rectum and bladder was twice observed, once independently of any other paralysis, and once as a part of a general affection. Amaurosis was noticed once, without any evidence of albuminuria.

Paralytic symptoms appeared ordinarily from the fourth to the eighth day; sometimes dysphagia was observed from the first; this symptom, however, we should hardly regard as conclusive evidence of paralysis, especially in such young subjects. In other cases the symptoms manifested themselves at a very advanced stage. The mean duration of the paralysis was one month, subject, however, to the chance of prolongation and aggravation by intercurrent affections. The treatment recommended by M. Roger is the same as that of previous observers, namely, tonics, iron, sulphur and electricity. We have seen no statement by M. Roger of the proportion of recoveries to deaths, but the experience of other observers justifies the most confident favorable prognosis even when the symptoms are of a very grave character. We have gathered the above facts of M. Roger's observations from the *Gazette des Hopitaux*."

Charcoal Ventilators for Sewers.—A rather important Report on the results of applying Charcoal to the Sewer Ventilators, in order to deodorize the fumes arising from them, has been made to the city of London Commissioners of Sewers by Dr. Letheby and Mr. Haywood. We need not tell our readers that there are miles of sewers under the London streets, and that the vapors arising from their decomposing contents, aggravated in too many cases by the bad condition of the sewers, which allows stagnation and decay, must be permitted to escape into the open air. Otherwise they would pass into private houses even more pertinaceously than they do at present, and would suffocate the unhappy sewer-man. The ventilating apertures permitting the escape of these gases are very numerous, and in some situations they give issue to a quantity sufficient to be a serious nuisance to the neighboring houses. They form a constant, and we fear increasing, element in that which constitutes the difference between town air and country air. The absorbing and oxodizing properties of charcoal, which has been much experimented on by Dr. Steinhouse, have caused it to be tried for the purpose of destroying these gases; "for," said Dr. Letheby three years ago, "let the gases go out of the sewers how they will, and where they will, you have but to place a small box containing a few pennyworths of charcoal in the course of the draught, and the purification of the air will be complete." In order to test the virtues of charcoal fairly, a space of fifty-nine acres in the city of London, near the minories, was selected. It has 1700 houses, 14,000 inhabitants, the sewers have but a slight fall and and are sluggish, and the streets are narrow, so that any emanations are doubly dangerous.

"The total length of sewers in the district is 25,587 feet; of which 2081 feet are pipes, and the remainder are constructed of brick, varying from 3 feet high by 2 feet wide to 5 feet high by 3 feet wide, internal dimensions."

Air filters, consisting each of a set of six trays filled with 6½ lbs. of wood charcoal broken into pieces the size of a filbert, were affixed to each of the ventilators on July 14, 1860, and have been in operation ever since with results which the reporter describes thus:—

"First. The deodorizing power of the charcoal has been satisfactorily proved to be complete. Not only have there been no complaints from the public of stenches from the ventilating gratings, but we have ascertained by actual observation that the odor of the sewer gases is not perceptible when they have traversed the chaarcoal." The charcoal employed is proved to contain abundance of alkaline nitrate, and peculiar alkaline salts and volatile bodies which may be described as the essence of stench. Secondly, the duration of the powers of the charcoal is uncertain, but if exposed to wet it requires to be changed frequently. Thirdly, and this is a point we desire to call attention to, it is evident that these charcoal fibres may hinder the escape of effluvia, not by deodorizing the currents of air which pass through them, but by hindering those currents from ascending; and in this case, although they may remedy one nuisance, and the charcoal may absorb some ill-smelling bodies, yet they really do not really answer the durpose of ventilation. And the reporters show that they have been unable to detect "any perceptible currents of air through the ventilators filled up with charcoal sieves. They allow of diffusion, but cannot be proved to allow of currents. There is no evidence that the air within the sewers was worse than before, or that the ventilators caused more sewer gas to enter private dwellings, but *there is evidence that these air filters act as ventilators.* Fourthly, the cost. The prime cost of the 104 ventilators fitted up within the aforesaid district was £918 18s. 5d., with a further annual charge of £131 5s. 4d., or £1 5s. 3d. each, for repairs and management. Considering the thousands of ventilating shafts in London, the expense becomes a very serious matter. Would it not be better to expend the money in improved construction, so as to hurry on the sewer stream, and diminish the evolution of gases, and to avoid these most expensive palliatives, for such they are?—*Lancet.*

---

Aconite and Nux Vomica Antidotal to each other.—In the case of a negro boy who had swallowed a destructive dose of the tincture of aconite and was rapidly sinking in spite of emetics and external irritants, Dr. D. D. Hanson administered three drops of the tincture of nux vomica. In a few minutes the heart's impulse returned with accelerated vigor, and the respirations were correspondingly improved in steadiness and and depth. After a repeated dose, twenty minutes later, vigorous emesis followed the tickling of the fauces with a feather. The next morning the patient was fully recovered. Dr. Woakes instituted during the last year a series of experiments with a view of ascertaining the applicability of aconite in strychnia-poisoning, from which he draws the following conclusions:—

1. In cases of poisoning by strychnia, it is the violence of the spasms which is the chief source of danger, the patient usually dying from the immediate effect of these, rather than from the exhaustion consequent upon the attack. It is the peculiar action of aconite to diminish the intensity, as well as to shorten the duration of these spasmodic contractions.

2. The influence of aconite in this respect is very transitory, requiring the dose to be cautiously repeated, according to the urgency of the symptoms.

3. Nevertheless, we are not to regard strychnia and aconite in the light of bane and antidote, as the only antagonism between them in a physiological, and not a chemical one, the contractile power of the muscles being strongly excited by the one, and powerfully relaxed by the other.

4. From this fact alone, it is probably impossible so to adjust the dose of each drug that both may be administered simultaneously, and the balance of the system remain undisturbed.

5. While the antagonism above alluded to is taking place, if the system be properly supported by stimulants, etc., strychnia will be gradually eliminated from it, and so the cure completed.

It remains to test the accuracy of these conclusions by observations in the human subject."

---

Asthma.—It would appear that a lady, a pupil at the Maternity of Paris, has just discovered a remedy for asthma. It consists in painting the chest with iodine pigment, and kneading the thorax in a peculiar manner. The remedy was tried in the Hospital of Tours, with the consent of the physicians, and found efficacious.—*London Lancet.*

The Blane Medals.—Two gold medals, founded by the late Sir Gilbert Blane, have just been awarded to Christopher Knox Ord, M. D., of H. M's ship *Hermes*, and to Mr. William Macleod, of H. M's ship *Madagascar.*—*Ibid.*

Desire for Information in Spain.—Dr. Francis Medina, chief physician of the Spanish Navy, has just been entrusted by the government of his country, with a very important mission. He is to undertake a series of travels to ascertain the sanitary state of the principle civilized countries.—*Ibid.*

Physicians and Surgeons.—Up to the present time, the physicians and surgeons of the General Hospital of Lisbon were higher in rank than the surgeons attached to the same institution. By a recent decree, this state of things has ceased to exist, and a footing of equality is now the rule.—*Ibid.*

